# AtRALF1 precursor processing in the Golgi complex involves two subtilisin-like serine proteases

**DOI:** 10.1007/s00425-026-05213-6

**Published:** 2026-11-03

**Authors:** Juan Carlos Guerrero-Abad, Tábata Bergonci, Marcio C. Silva-Filho, Daniel S. Moura

**Affiliations:** 1 Departamento de Ciências Biológicas, Escola Superior de Agricultura Luiz de Queiroz, ESALQ, Universidade de São Paulo, USP, Piracicaba, SP 13418-900 Brazil; 2 Departamento de Genética, Escola Superior de Agricultura Luiz de Queiroz, ESALQ, Universidade de São Paulo, USP, Piracicaba, SP 13418-900 Brazil; 3 Present Address: Instituto de Investigación, Facultad de Ingeniería y Ciencias Agrarias, Innovación y Desarrollo Para El Sector Agrario y Agroindustrial (IIDAA), Universidad Nacional Toribio Rodríguez de Mendoza de Amazonas, Chachapoyas, 01000 Peru; 4 Present Address: Institut for Retsmedicin, Aarhus University, AU, Aarhus N, 8200 Aarhus, Denmark

**Keywords:** Rapid alkalinization factor, Subtilase, Prohormone processing, Small peptides, Lateral root density, Golgi complex

## Abstract

**Main conclusion:**

AtSBT6.1 is the primary physiological convertase for AtRALF1, while AtSBT5.3 contributes under ectopic AtRALF1 overexpression.

**Abstract:**

The 5-kDa rapid alkalinization factor (RALF) peptide is widespread in the plant kingdom. In Arabidopsis, the RALF family comprises 37 RALF-like peptides. AtRALF1 is a 49-amino-acid peptide derived from a 120-amino-acid precursor, but the protease responsible for its maturation has remained unresolved. We used in silico expression profiling to select subtilases (AtSBTs) potentially involved in AtRALF1 precursor processing. T-DNA insertion AtSBT mutants, and F3 homozygous lines derived from crosses between AtSBT mutants, and an AtRALF1-overexpressing line were analyzed for primary root length (PRL) and lateral root primordium (LRP) density. PRL and LRP density were higher in atsbt6.1 than in wild-type plants, whereas atsbt5.3 was indistinguishable from the wild-type. Ectopic expression of AtRALF1 driven by the 35S promoter in atsbt6.1 and atsbt5.3 backgrounds indicated that both AtSBT6.1 and AtSBT5.3 contribute to AtRALF1 precursor processing under overexpression conditions. In contrast, the phenotypes of single mutants indicate that AtSBT6.1 plays the predominant role in AtRALF1 precursor processing when AtRALF1 is expressed from its endogenous promoter. Transient expression assays further showed that AtRALF1 precursor processing occurs in the Golgi complex. These findings identify AtSBT6.1 as the main subtilase for AtRALF1 maturation in planta and reveal context-dependent participation of AtSBT5.3 under ectopic AtRALF1 expression.

**Supplementary Information:**

The online version contains supplementary material available at https://doi.org/10.1007/s00425-026-05213-6.

## Introduction

Rapid alkalinization factor (RALF) is a family of small, secreted plant peptide hormones that are involved in the regulation of plant growth, development, reproduction, immunity, and responses to environmental stimuli (Pearce et al. 2001; Haruta et al. 2008; Mingossi et al. 2010; Covey et al. 2010; Stegmann et al. 2017; Ge et al. 2017; Zhao et al. 2018). Since their initial characterization as extracellular alkalinization signals, RALF peptides have emerged as signaling ligands that coordinate cell wall integrity sensing and receptor kinase activation in diverse biological contexts (Blackburn et al. 2020; Cheung and Wu 2024). In Arabidopsis, RALF peptides (AtRALFs) regulate several developmental processes including root growth, hypocotyl elongation, lateral root density, pollen tube growth, cell wall integrity, and immune responses (Morato do Canto et al. 2014; Mang et al. 2017; Qu et al. 2017; Gonneau et al. 2018).

The AtRALF peptides are recognized mainly by *Catharanthus roseus* RLK1-like receptor kinases, including FERONIA, ANXUR and THESEUS1-related receptors, often together with LORELEI-like GPI-anchored proteins (Ge et al. 2017; Mang et al. 2017; Gonneau et al. 2018; Cheung and Wu 2024). Their signaling triggers rapid cellular responses such as extracellular alkalinization through regulation of plasma membrane H+-ATPase activity, Ca^2^⁺ mobilization, and modulation of reactive oxygen species (Morato do Canto et al. 2014; Haruta et al. 2014; Feng et al. 2019).

Many secreted peptide ligands are synthesized as biologically inactive prepropeptides that enter the conventional secretory pathway, where the signal peptide is removed and the remaining propeptide undergoes structural modification and proteolytic processing to generate the mature bioactive ligand (Matsubayashi 2011; Tavormina et al. 2015; Furumizu and Shinohara 2024). RALF precursors contain an N-terminal signal peptide, and RALF homologues from *Nicotiana benthamiana* and other species initially localize to the endoplasmic reticulum and later to the cell wall when expressed as GFP fusions (Escobar et al. 2003). In addition to the signal peptide, NtRALF and several homologues contain a double-arginine dibasic site upstream of the N-terminus of the mature peptide, suggesting that maturation requires proteolytic cleavage by convertase-like enzymes, analogous to prohormone processing systems in animals and yeast (Seidah and Chretien 1999; Pearce et al. 2001).

Transgenic plants overexpressing AtRALF1 are semi-dwarf and this phenotype is abolished when the second arginine of the dibasic site upstream of the mature peptide is replaced by alanine (AtRALF1R69A) (Matos et al. 2008). Similarly, AtRALF23 overexpression reduces plant growth; wild-type phenotypes are restored when the conserved dibasic site in the AtRALF23 precursor is replaced by two glycines (RR to GG) or when AtRALF23 overexpression is introgressed into the *atsbt6.1* background, which is defective in the subtilisin-like serine protease AtSBT6.1 (Srivastava et al. 2009). These results provide genetic evidence that subtilisin-like proteases are involved in the maturation of at least some RALF precursors.

In Arabidopsis, subtilases comprise a family of 56 genes divided into six subfamilies involved in protein turnover, seed development, cell wall rearrangement, programmed cell death, and peptide hormone processing (Rautengarten et al. 2005; Schaller et al. 2012). In addition to processing AtRALF23, several subtilases have been implicated in the maturation of peptide ligands such as IDA, CLE-related peptides, RGF/GLV/CLEL peptides and PSK (Schardon et al. 2016; Ghorbani et al. 2016; Stührwohldt et al. 2020a; Royek et al. 2022). Protease–substrate relationships in plants are often highly dynamic: a single subtilase may process multiple peptide precursors, whereas one precursor may require more than one protease for full maturation. Moreover, substrate specificity depends not only on dibasic motifs but also on extended sequence context, post-translational modifications, subcellular localization, and pH conditions (Schardon et al. 2016; Stuhrwohldt et al. 2021). Despite these advances, the molecular mechanisms controlling AtRALF1 precursor maturation, and the identity of the proteases responsible under physiological conditions, remain incompletely understood.

To identify the subtilase responsible for AtRALF1 precursor processing, we selected seven Arabidopsis subtilases as candidate processing enzymes and introgressed the AtRALF1-overexpression transgene (p35S:AtRALF1) into each mutant background. The evaluation of homozygous lines derived from these crosses showed that AtSBT5.3 and AtSBT6.1 are involved in AtRALF1 precursor processing in p35S:AtRALF1 lines. The comparison of subtilase mutant phenotypes indicated that AtSBT6.1 is responsible for peptide processing under physiological conditions, whereas both AtSBT6.1 and AtSBT5.3 can process the AtRALF1 precursor when AtRALF1 is expressed under the constitutive 35S promoter. Using transient expression and the Golgi retention domain of beta-1,2-XYLOSYLTRANSFERASE, we further demonstrated that AtRALF1 precursor processing occurs in the Golgi apparatus.

## Material and methods

### In silico analysis

Gene expression datasets available from The Arabidopsis Information Resource (www.arabidopsis.org) and the Arabidopsis eFP Browser (http://bar.utoronto.ca/efp/cgi-bin/efpWeb.cgi) were used to analyze the expression of the 56 Arabidopsis subtilases, as described by Rautengarten et al. (2005).

### Plant material and growth conditions

Wild-type *Arabidopsis thaliana* seeds (ecotype Col-0), p35S:AtRALF1 plants (Matos et al. 2008) and the T-DNA insertion lines *atsbt5.3* (SALK_125788), *atsbt1.4* (SALK_147962), *atsbt2.6* (SALK_068944), *atsbt4.12* (SALK_045125), *atsbt1.8* (SALK_020799), *atsbt6.1* (SALK_020530) and *atralf1-1* (SALK_036331) were obtained from the Arabidopsis Biological Resource Center, Ohio State University, Columbus, USA. F3 progenies were surface-sterilized and stratified at 4 °C for 3 days in sterile water. Seeds were germinated on vertical plates containing half-strength MS medium without sucrose, 0.6% phytagel, pH 5.7. The plates were maintained in growth chambers at 22 °C, 70% relative humidity, 450 µmol m-2 s-1 and a 16 h/8 h photoperiod.

### Root growth inhibition assay

The seeds of Col-0, *atsbt5.3* (SALK_125788), and *atsbt6.1* (SALK_020530) were germinated on vertical plates containing half-strength MS medium without sucrose, supplemented with 0.6% phytagel and adjusted to pH 5.7. Ten-day-old seedlings were transferred to fresh plates containing the same medium either without AtRALF1 (control) or supplemented with 5 µM AtRALF1. Recombinant AtRALF1 peptide was obtained as previously described (Morato do Canto et al. 2014). The primary root length was measured at 24, 48, 72, and 96 h after transfer. Root length measurements were performed using ImageJ software.

### Plasmid construction and chimeric fusions

Full-length cDNAs encoding *AtSBT5.3*, *AtSBT6.1*, and *AtSBT1.7* were amplified using gene-specific primers (Supplementary Table S2) and standard PCR conditions. The fragments were purified, cloned into the Gateway entry vector pENTR/D-TOPO (Life Technologies) and transferred by recombination into the destination vectors pK7FWG2 (*AtSBT5.3*, *AtSBT6.1* and *AtRALF1*) and pK7GWIWG2I (*AtSBT1.7*) (Karimi et al. 2002). The chimeric fusions for Golgi retention were constructed by replacing the AtRALF1 signal peptide with the first 35 amino acids of the β-1,2-XYLOSYLTRANSFERASE gene (At5g55500; *AtXylT35*), a Golgi-resident targeting sequence (Pagny et al. 2003). The AtRALF1 coding region, with or without the R69A mutation, was amplified from Col-0 genomic DNA or from p35S:AtRALF1(R69A) transgenic plants (Matos et al. 2008). The p35S:AtRALF1-GFP plasmid (pK7FWG2) was used as a template to add a KDEL ER-retention sequence to the C-terminus of GFP (p35S:AtRALF1-GFP-KDEL). All fragments were cloned into pENTR/D-TOPO and recombined into pK7FWG2 [AtXylT35-proAtRALF1 and AtXylT35-proAtRALF1(R69A)] or pK7WG2 (preproAtRALF1-GFP-KDEL). A chimeric fusion between the AtRALF1 precursor and monomeric mCherry under the control of the 35S promoter was also constructed. The T35S terminator, mCherry and AtRALF1 sequences were amplified separately from pK7FWG2 (Karimi et al. 2002), pAN583 (Shaner et al. 2004) and genomic DNA, respectively. These fragments were reconstituted as a single AtRALF1-mCherry-T35S fragment (1300 bp) using gene-specific primers (Supplementary Table S2) and inserted between the ApaI and SpeI sites of pK7FWG2.

### Transgenic plants and transient expression assays

*Agrobacterium tumefaciens* strain GV3101 carrying the vectors of interest (pK7FWG2, pK7WG2 and pK7GWIWG2I) was used to generate transgenic plants by the floral dip method (Clough and Bent 1998). The seeds from transformed plants were plated on a half-strength MS medium without sucrose containing 100 mg l-1 kanamycin (Sigma). Arabidopsis T3 seeds were used in all experiments. For transient expression assays in *Nicotiana benthamiana* leaves, *A. tumefaciens* carrying the vectors of interest was grown for 48 h at 28 °C with shaking at 180 rpm. Bacterial cultures (1 mL) were harvested, centrifuged (5000 rpm, 5 min) and resuspended in 1 mL infiltration buffer (10 mM MgCl2, 10 mM MES, pH 5.7, and 200 µM acetosyringone) for 4 h at room temperature. At 2–5 days after infiltration, the leaves were analyzed by confocal microscopy or immunoblotting.

### Immunoblot analysis

Transiently transformed *N. benthamiana* leaves were subjected to total protein extraction in extraction buffer (20 mM Tris–HCl, pH 7.5, 150 mM NaCl, 1 mM EDTA, 1% Triton X-100, 0.1% SDS, 5 mM DTT, 1 mM PMSF, 10 µM leupeptin, 1 µM pepstatin and 0.3 µM aprotinin). After centrifugation (20,000 g, 15 min, 4 °C), an aliquot was used for protein quantification according to Bradford (1976). The aliquots of each supernatant were resolved on 15% SDS-PAGE gels, and the proteins were detected by immunoblotting using anti-AtRALF1 antiserum (1:5000) as the primary antibody and alkaline phosphatase-conjugated anti-rabbit IgG (whole molecule; 1:15,000, BIORAD, Cat. Number 1706518) as the secondary antibody. Anti-AtRALF1 rabbit antibodies were produced by CelulaB, UFRGS (Brazil).

### Semi-quantitative RT-PCR and quantitative qRT-PCR analysis

Total RNA was isolated from roots of 8-day-old Arabidopsis plants grown on vertical plates using TRIzol reagent according to the manufacturer’s instructions (Life Technologies). cDNA (1 µL) was used as a template for RT-PCR with gene-specific primers (Supplementary Table S2). qRT-PCR analysis was performed using 10-fold diluted cDNA, Maxima SYBR Green/ROX qPCR Master Mix (Thermo Scientific) and a StepOne Real-Time PCR System (Applied Biosystems). The primers are listed in Supplementary Table S2. GLYCERALDEHYDE 3-PHOSPHATE DEHYDROGENASE (GAPDH) was used as an internal control for both RT-PCR and qRT-PCR. Three technical replicates were analyzed for each biological sample, together with a no-template control. At least three independent biological samples were evaluated. The cycle threshold (Ct) was determined automatically by the instrument, and relative gene expression was calculated using the 2-ΔΔCt method (Livak and Schmittgen 2001). An arbitrary value of 1 was assigned to wild-type plants. The graphs show one representative biological replicate.

### Confocal microscopy

The transgenic seedlings and leaves transiently expressing chimeric proteins were visualized using an Olympus FV1000 confocal microscope. GFP and mCherry were excited at 488 and 587 nm, respectively, and emission was measured at 500–530 nm and 610 nm. The images were analyzed using the Olympus FV1000 software and ImageJ (https://imagej.nih.gov/ij/).

### Lateral root primordium density

The roots were cleared as described by Malamy and Benfey (1997) and lateral root primordia and total lateral root primordia were quantified using a differential interference contrast microscope (BX51; Olympus).

### Crossing procedures and analysis of the hybrids

p35S:irAtSBT1.7 and SALK T-DNA insertion lines were used as female parents, and p35S:AtRALF1 or p35S:AtRALF1-mCherry lines were used as male parents. F2 and F3 progenies were selected by semi-quantitative RT-PCR and fluorescence detection (165FC; Leica).

### Statistical analyses

Statistical analyses were performed using Infostat Statistics Base software (version 2017e). The means were compared by analysis of variance followed by Tukey’s honestly significant difference test (Steel et al. 1997). Scatter dot plots were generated using GraphPad Prism version 6.0.

## Results

### Selection of putative subtilisin-like serine proteases involved in AtRALF1 preproprotein processing based on mRNA abundance in Arabidopsis roots

**Fig. 1:**
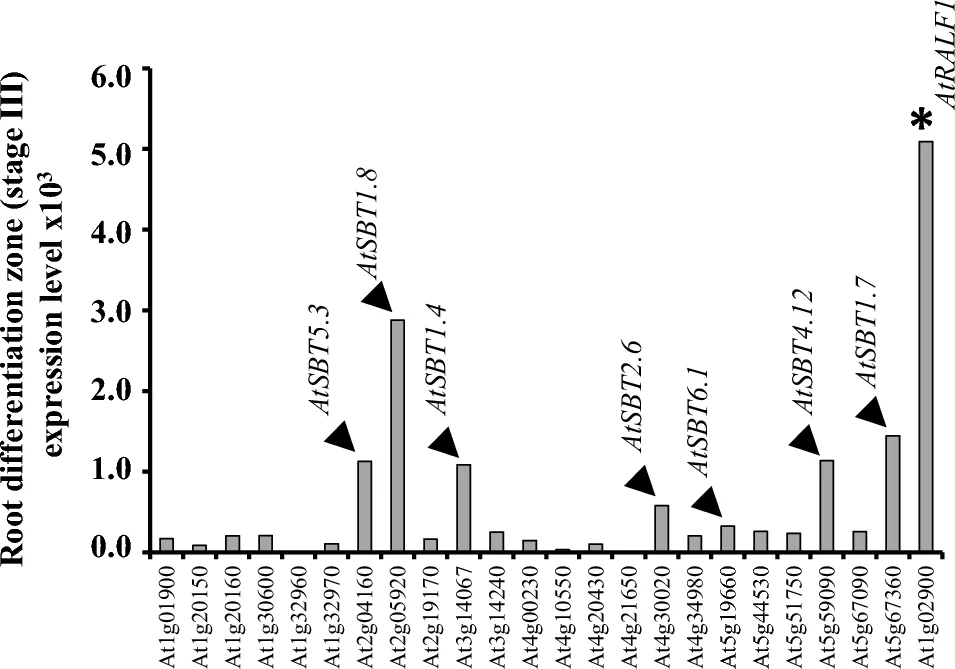
In silico gene expression profiles of 23 subtilisin-like serine proteases (subtilases; *AtSBTs*) in the root differentiation zone (stage III) of Arabidopsis plants according to the Arabidopsis eFP Browser (Birnbaum et al. 2003). The arrowheads indicate highly expressed subtilases selected for further analysis. *AtRALF1* gene expression is shown for comparison (asterisk)

Of the 56 Arabidopsis subtilases, 46 are predicted to be targeted to the secretory pathway (Rautengarten et al. 2005). Because *AtRALF1* is predominantly expressed in roots, we first excluded subtilases not expressed in root tissues. According to in silico data, 23 of the 46 secreted subtilases are expressed in roots. In root tissues, *AtRALF1* is mainly expressed in the root differentiation zone. To narrow the number of putative AtRALF1-processing proteases, we used available gene expression data and the eFP Browser (Winter et al. 2007) to select subtilases expressed in the root differentiation zone (stage III; Fig. 1). Among the 23 root-expressed subtilases, those with the highest mRNA levels in the differentiation zone were selected for further investigation: *AtSBT5.3*, *AtSBT1.8*, *AtSBT1.4*, *AtSBT2.6*, *AtSBT6.1*, *AtSBT4.12*, and *AtSBT1.7* (Fig. 1, arrowheads). We then confirmed the expression of these seven subtilases in Arabidopsis roots by semi-quantitative PCR. Overall, the results reflected the relative mRNA levels predicted from in silico data (Fig. 1). AtSBT1.8 mRNA accumulated at the highest level, whereas AtSBT6.1 accumulated at the lowest level. The differences between the in silico and semi-quantitative results may reflect the fact that the in silico analysis focused on the differentiation zone, whereas the semi-quantitative analysis used whole roots. We also evaluated subtilase gene expression in plants overexpressing AtRALF1 (Matos et al. 2008). The mRNA accumulation levels of the seven subtilases were not altered in p35S plants (Supplementary Fig. S1), suggesting that AtRALF1 does not regulate the expression of its precursor-processing enzyme(s).

### The short-root phenotype caused by AtRALF1 gene overexpression is dependent on AtSBT6.1 and is partially affected by the absence of AtSBT5.3

Plants with short roots and a semi-dwarf phenotype are produced when *AtRALF1* is expressed under the control of the 35S promoter (p35S:AtRALF1; Matos et al. 2008). We reasoned that crossing p35S:AtRALF1 plants with mutants defective in the subtilase responsible for AtRALF1 processing would mitigate the overexpression phenotype despite high *AtRALF1* transcript accumulation. To test this, T-DNA insertion mutants for the seven selected subtilases were crossed with p35S:AtRALF1 plants. For AtSBT1.7, for which no T-DNA mutant was available, we used homozygous lines in which *AtSBT1.7* was silenced by an inverted-repeat construct (*irAtSBT1.7*). The T-DNA insertion sites, semi-quantitative gene expression analysis, validation of T-DNA insertions and representative images of each mutant or ir-line are shown in Supplementary Fig. S2. F2 plants homozygous for the p35S:AtRALF1 transgene and for the corresponding T-DNA insertion were selected by PCR and self-fertilized to generate F3 seeds. Forty F2 plants were evaluated for each cross, and at least two plants from each cross were confirmed as homozygous for both loci (Supplementary Table S1).

**Fig. 2:**
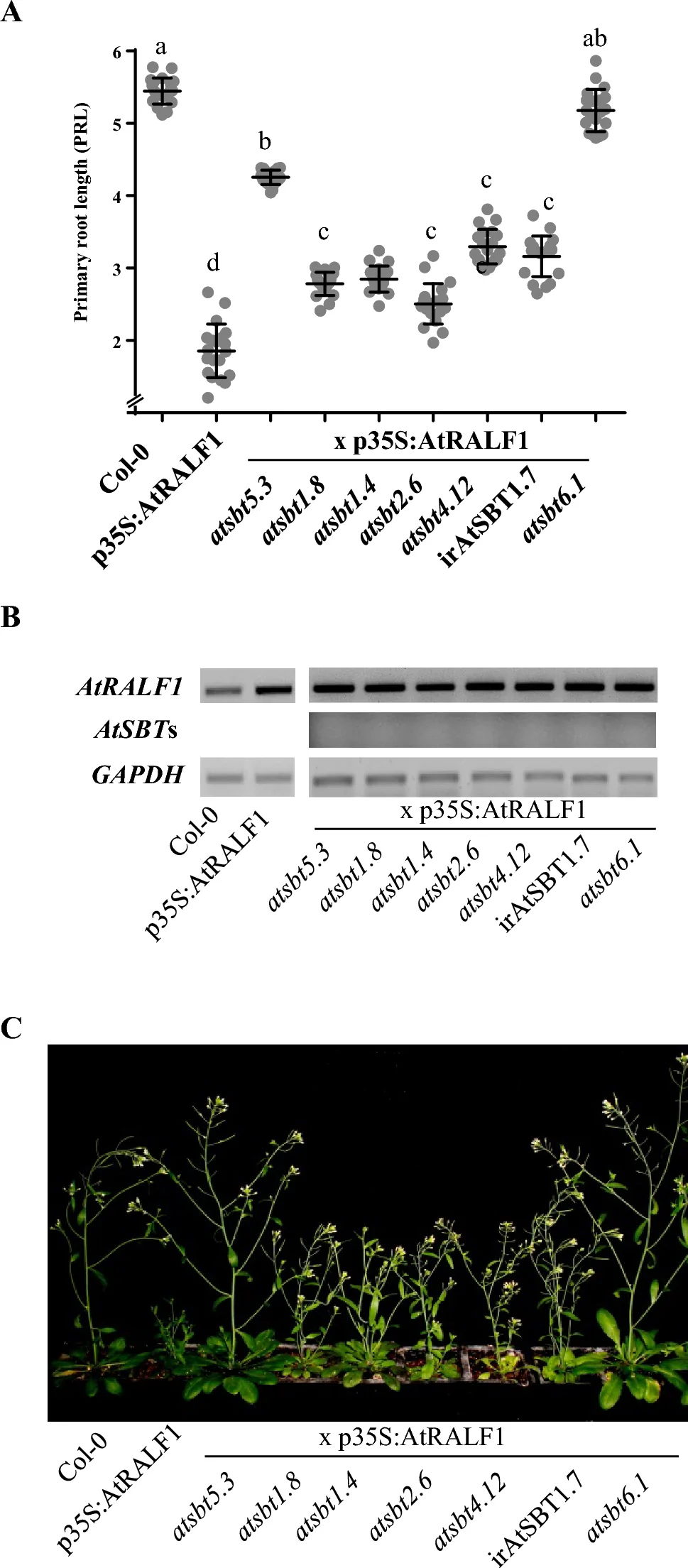
Analysis of F3 progenies derived from crosses between the AtRALF1-overexpressing line (p35S:AtRALF1) and subtilisin-like serine protease (subtilase; *atsbt*) T-DNA insertion mutant lines. For AtSBT1.7, an inverted-repeat transgenic line (*irAtSBT1.7*) was used instead of a T-DNA line. **A** Primary root length of plants grown on vertical plates for 10 days. Data points with the same letter are not significantly different (Tukey test, *P* < 0.01; *n* = 20). **B** Semi-quantitative RT-PCR analysis of *AtRALF1* and *AtSBT* genes. Total RNA was extracted from roots of 10-day-old homozygous plants. RT-PCR was performed using 28 cycles, and *GAPDH* was used as an internal control. **C** Representative images of Col-0, p35S:AtRALF1 and F3 plants grown on soil for 45 days. Col-0, wild-type

Primary root length analysis of F3 seedlings derived from these crosses showed that p35S:AtRALF1 plants recovered wild-type root growth in the *atsbt6.1* background and partially recovered root growth in the *atsbt5.3* background (Fig. 2A). All other F3 crossed lines, including *irAtSBT1.7*, showed reduced root growth, suggesting that AtRALF1 processing is specifically impaired in the *atsbt6.1* and *atsbt5.3* backgrounds. Semi-quantitative RT-PCR of homozygous F3 seedlings confirmed *AtRALF1* transcript accumulation and the absence of the corresponding subtilase transcript (Fig. 2B). The characteristic semi-dwarf phenotype of p35S:AtRALF1 plants was not observed in p35S:AtRALF1/*atsbt5.3* or p35S:AtRALF1/*atsbt6.1* F3 plants (Fig. 2C). Together, primary root growth and mature-plant phenotypes indicate that AtSBT6.1 and AtSBT5.3 are involved in AtRALF1 precursor processing under *AtRALF1* overexpression conditions. We also evaluated the sensitivity of root growth to exogenously applied recombinant AtRALF1 peptide (5 µM) in the *atsbt5.3* and *atsbt6.1* mutant lines, as well as in Col-0 plants. All genotypes were sensitive to AtRALF1 treatment, exhibiting a significant reduction in root growth compared with their respective untreated controls (Supplementary Fig. S3A–B).

### Reduction of lateral root density in p35S:AtRALF1 seedlings is dependent on AtSBT5.3 and is partially affected by AtSBT6.1

To refine the analysis of AtRALF1 processing and the specific roles of AtSBT5.3 and AtSBT6.1, we generated an additional p35S:AtRALF1 event for crosses with *atsbt6.1* and *atsbt5.3* mutants. To facilitate the selection of transformed and segregating F2 seedlings and to detect the AtRALF1 fusion protein, the AtRALF1 precursor was fused to the mCherry reporter protein (Nelson et al. 2007). Eight independent transgenic events with high AtRALF1-mCherry expression were obtained. All showed the same phenotypic characteristics, and one event was quantitatively evaluated. p35S:AtRALF1-mCherry plants showed short roots and reduced lateral root density, as previously observed for p35S:AtRALF1 plants (Matos et al. 2008; Supplementary Fig. S4A, B). We also tested whether the AtRALF1-upregulated genes *CONSTITUTIVE PHOTOMORPHISM AND DWARFISM* (*CPD*) and *HYDROXYPROLINE-RICH GLYCOPROTEIN2* (*AtHRGP2*), and the AtRALF1-downregulated gene *EXPANSIN A5* (*AtEXPA5*), responded accordingly in p35S:AtRALF1-mCherry plants. All three genes responded as expected and similarly to their responses in the original p35S:AtRALF1 event (Bergonci et al. 2014a, b; Supplementary Fig. S4C–E). AtRALF1-mCherry fluorescence was readily observed in seeds 48 h after germination, facilitating selection of p35S:AtRALF1-mCherry-overexpressing lines (Supplementary Fig. S4F). The p35S:AtRALF1-mCherry transgene was introduced into *atsbt6.1* and *atsbt5.3* backgrounds by crossing mutant genotypes with the transgenic line. The homozygous lines were selected by PCR using specific SALK T-DNA primers (Supplementary Table S2), and mCherry fluorescence was detected by stereomicroscopy. The *atralf1* null mutant carrying a T-DNA insertion in *AtRALF1* was also used in the experiments (Supplementary Fig. S5A, B).

**Fig. 3:**
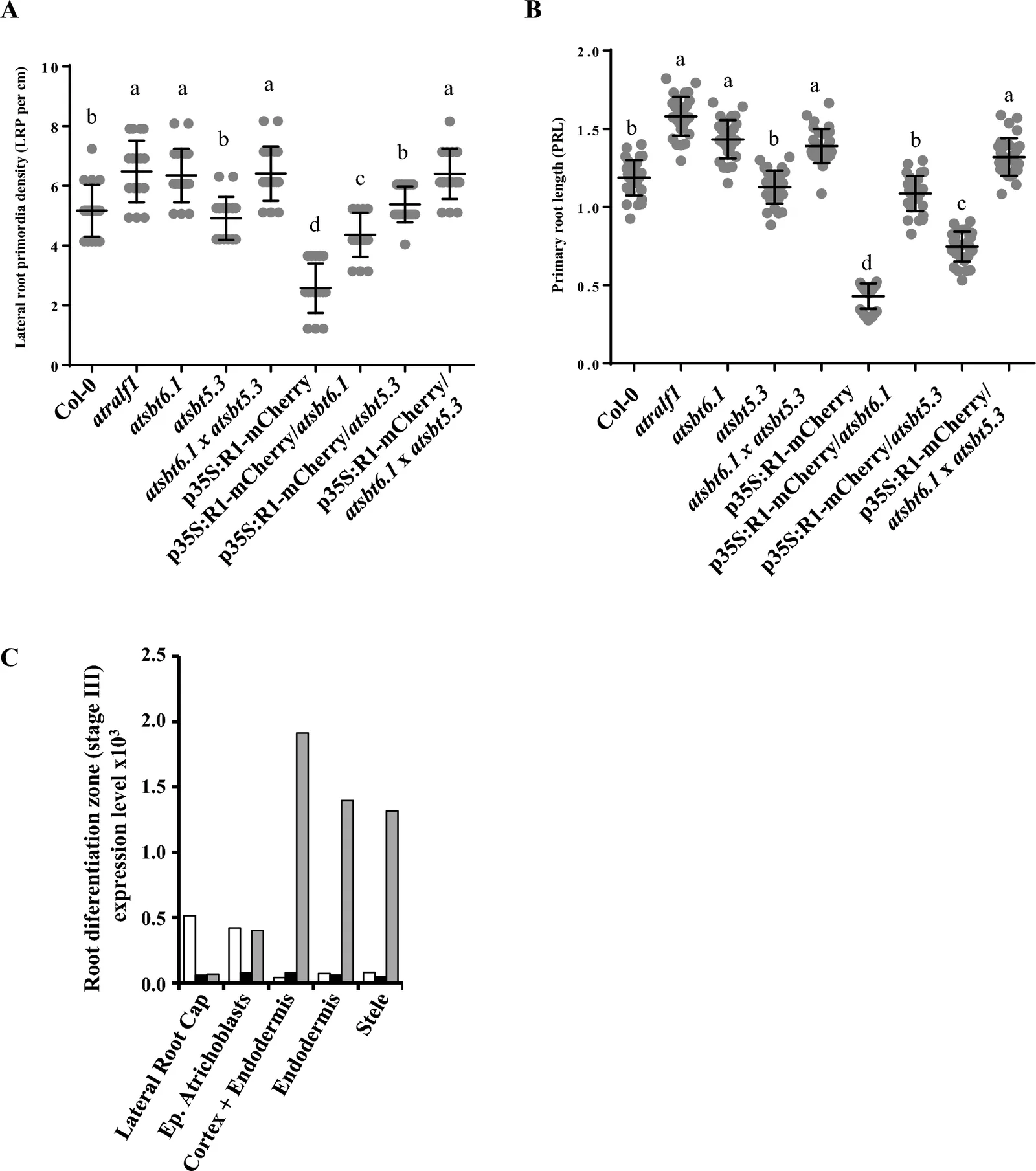
Characterization of the *atralf1* null mutant and F3 progenies derived from crosses between *AtRALF1*-overexpressing plants and *atsbt5.3* and *atsbt6.1* null mutants (p35S:R1-mCherry/atsbt6.1, p35S:R1-mCherry/*atsbt5.3* and p35S:R1-mCherry/*atsbt6.1* × *atsbt5.3*). **A** Lateral root primordium (LRP) density of seedlings grown on vertical plates for 5 days. Data points with the same letter are not significantly different (Tukey test, *P* < 0.01; *n* = 18). **B** Primary root length (PRL) of seedlings grown on vertical plates for 6 days. Data points with the same letter are not significantly different (Tukey test, *P* < 0.01; *n* = 30). **C** In silico expression of *AtSBT5.3*, *AtSBT6.1* and *AtRALF1* in the root differentiation zone (stage III) across different cell layers. White, black and gray bars represent *AtSBT5.3*, *AtSBT6.1* and *AtRALF1*, respectively

The analysis of lateral root primordium (LRP) density in mutants and F3 homozygous lines showed that *atsbt6.1* and *atralf1* had similar values, and both showed higher LRP density than wild-type plants. The p35S:AtRALF1-mCherry/*atsbt6.1* line showed intermediate LRP density between the parental *atsbt6.1* and p35S:AtRALF1-mCherry lines. This pattern differed from that observed for *atsbt5.3* and p35S:AtRALF1-mCherry/*atsbt5.3*, both of which exhibited LRP density values like those of wild-type plants (Fig. 3A). These results indicate that AtSBT6.1 is involved in AtRALF1 processing when AtRALF1 is expressed from its native promoter but is not solely responsible when AtRALF1 is expressed from the 35S promoter. The lack of a phenotype in *atsbt5.3* suggests that AtSBT5.3 does not play a major role in AtRALF1 precursor processing under native AtRALF1 expression. However, under 35S-driven expression, AtSBT5.3 appears to have a prominent role in processing the AtRALF1 proprotein. Recovery of the *atralf1*-like LRP phenotype in the p35S:AtRALF1-mCherry/*atsbt6.1* × *atsbt5.3* F3 line confirms that both subtilases contribute to AtRALF1 precursor processing in overexpression lines.

### The short-root phenotype caused by AtRALF1 gene overexpression is dependent on AtSBT6.1 and is partially affected by the absence of AtSBT5.3

We also compared primary root length (PRL) among F3, mutant, parental and wild-type lines. The primary roots of *atsbt6.1* and *atralf1* mutants were longer than those of wild-type plants, and p35S:AtRALF1-mCherry/*atsbt6.1* plants had PRL values indistinguishable from those of wild-type plants (Fig. 3B). The *atsbt5.3* mutant had PRL values like those of wild-type plants, whereas F3 plants derived from p35S:AtRALF1-mCherry/*atsbt5.3* showed intermediate PRL values. These data indicate that, when AtRALF1 is expressed from its native promoter, AtSBT6.1 is probably the primary subtilase responsible for AtRALF1 proprotein processing. For PRL, AtSBT6.1 also appears to be the main subtilase responsible for precursor processing under 35S-driven AtRALF1 expression. AtSBT5.3 does not affect PRL under native AtRALF1 regulation, but under ectopic AtRALF1 expression it appears to share preproAtRALF1 processing with other subtilases, probably AtSBT6.1. Indeed, AtRALF1 overexpression in the *atsbt6.1* × *atsbt5.3* double-mutant background mimicked the atralf1 mutant phenotype. We then examined the in silico expression profiles of *AtRALF1*, *AtSBT6.1* and *AtSBT5.3* in roots. Based on the Arabidopsis root gene expression map (Birnbaum et al. 2003), *AtRALF1* is expressed mainly in the endodermis under its native promoter. Although both *AtSBT6.1* and *AtSBT5.3* are also expressed in the endodermis, *AtSBT5.3* is expressed mainly in the epidermis and lateral root cap cells (Fig. 3C).

### Processing of the AtRALF1 precursor occurs in the Golgi complex

**Fig. 4:**
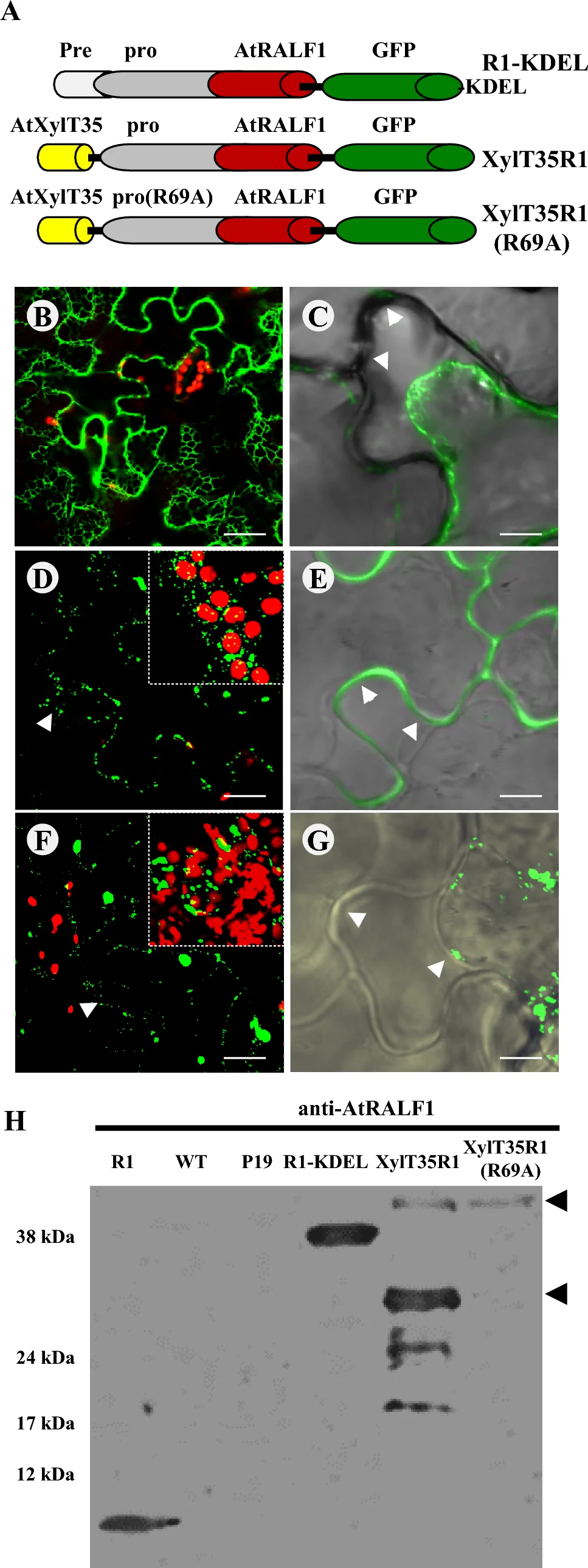
AtRALF1 precursor is processed in the Golgi complex. **A** Schematic diagram of constructs used to generate chimeric proteins carrying the AtRALF1 precursor (proAtRALF1) fused to green fluorescent protein (GFP), with or without the signal peptide (Pre), the endoplasmic reticulum (ER) retention domain (KDEL), or the Golgi retention domain (AtXylT35), or a mutation in the proAtRALF1 sequence [pro(R69A)]. **B**–**G** *N. benthamiana* transient expression of the constructs shown in **A**. Arrowheads indicate sites where the plasma membrane was pulled away from the cell wall. Bars = 10 µm. **B** PreproAtRALF1-GFP containing the ER-retention domain KDEL. **C** Plasmolyzed cell expressing preproAtRALF1-GFP-KDEL. **D** AtXylT35-proAtRALF1-GFP. **E** Plasmolyzed cell expressing AtXylT35-proAtRALF1-GFP. **F** AtXylT35-pro(R69A)AtRALF1-GFP. **G** Plasmolysed cell expressing AtXylT35-pro(R69A)AtRALF1-GFP. **H** Immunoblot probed with anti-AtRALF1. Total protein extracts from *N. benthamiana* leaves expressing preproAtRALF1-GFP-KDEL (39 kDa; R1-KDEL) or AtXylT35-proRALF1-GFP (42 kDa; XylT35R1) or AtXylT35-proRALF1(R69A)-GFP [42 kDa; XylT35R1(R69A)] were separated by SDS-PAGE. Leaves from untransformed plants (WT) and plants transformed with the P19 vector were used as negative controls. Recombinant AtRALF1 peptide (4 ng; R1) was used as a positive control. Arrowheads indicate unprocessed and processed AtRALF1 precursor products

Previously, we showed that the enzymatic activity responsible for AtRALF1 precursor processing is localized in the microsomal fraction (Matos et al. 2008). To define the subcellular site of AtRALF1 precursor processing, we generated AtRALF1 chimeric proteins (Fig. 4A). The first construct encoded preproAtRALF1 fused to GFP and carried the ER retention signal KDEL at the GFP C-terminus (preproAtRALF1-GFP-KDEL). The second construct encoded proAtRALF1-GFP fused at the N-terminus to the first 35 amino acids of Arabidopsis β-1,2-XYLOSYLTRANSFERASE (AtXylT35), a sequence used to retain proteins in the Golgi complex (Pagny et al. 2003). When transiently expressed in *N. benthamiana* leaves, preproAtRALF1-GFP-KDEL produced fluorescence in reticulate structures resembling the ER (Fig. 4B). After plasmolysis, the fluorescent signal moved away from the cell wall, indicating that the protein was retained in the ER and was not secreted (Fig. 4C). AtXylT35-proAtRALF1-GFP, designed to promote Golgi retention, was detected in Golgi-like punctate structures as well as in the cell wall (Fig. 4D, E). In contrast, AtXylT35-proRALF1(R69A)-GFP was not detected at the cell wall and was observed only in Golgi-like structures (Fig. 4F, G). We then prepared total protein extracts from infiltrated leaves to evaluate AtRALF1 processing by immunoblotting. Leaves infiltrated with preproAtRALF1-GFP-KDEL and probed with anti-AtRALF1 antibodies showed a band consistent with the expected 39-kDa proAtRALF1-GFP-KDEL protein, indicating that processing does not occur when the precursor is retained in the ER. However, protein extracts from leaves infiltrated with AtXylT35-proAtRALF1-GFP showed one band corresponding to the expected 42-kDa AtXylT35-proAtRALF1-GFP chimeric protein and a second band consistent with the expected 32-kDa AtRALF1-GFP product. Two additional low-molecular-weight bands were detected and likely represent AtRALF1-GFP degradation products. Finally, to confirm that the 32-kDa band resulted from proper processing of the proAtRALF1-GFP precursor, we analyzed protein extracts from the AtXylT35-proAtRALF1(R69A)-GFP system. In this case, the only band detected corresponded to the unprocessed 42-kDa AtXylT35-proAtRALF1(R69A)-GFP precursor (Fig. 4H). Together, confocal microscopy and immunoblot detection of AtRALF1 processing products mapped AtRALF1 precursor processing to the Golgi complex.

To determine whether the subtilases are targeted to the secretory pathway, we fused GFP to the C-terminus of AtSBT5.3 and AtSBT6.1. Each construct was placed under the control of the 35S promoter and stably integrated into the Arabidopsis genome. The primary root cells from independent T3 transgenic lines (p35S:AtSBT5.3-GFP, n = 3; p35S:AtSBT6.1-GFP, n = 9) and wild-type plants were visualized by confocal microscopy. All lines examined showed intense fluorescence at the cell periphery and within cells (Supplementary Fig. S6A-C). To distinguish cell-wall/apoplastic fluorescence from cytoplasmic or plasma-membrane fluorescence, the roots were plasmolyzed by incubation in 0.8 M mannitol for 30 min. After plasmolysis, the plasma membrane was displaced from the cell wall, whereas fluorescence remained detectable in cell walls, indicating that these subtilases are secreted to the apoplast (Supplementary Fig. S6D–F). In root cells of p35S:AtSBT5.3-GFP and p35S:AtSBT6.1-GFP lines, fluorescence was also observed in cytosolic punctate structures resembling Golgi bodies.

## Discussion

Genes encoding RALF precursors are expressed in a tissue-specific manner in Arabidopsis (Morato do Canto et al. 2014). Because AtRALF1 is secreted and expressed mainly in the root differentiation zone (Dressano et al. 2017), we selected seven of the 56 Arabidopsis subtilases as candidates for AtRALF1 proprotein processing.

After selecting seven candidate subtilases potentially responsible for AtRALF1 processing, we crossed each corresponding mutant with the AtRALF1-overexpressing line (p35S:AtRALF1). Our rationale was that progeny carrying mutations in subtilases required for AtRALF1 processing would display a wild-type-like phenotype even in the presence of high AtRALF1 expression. Only two of the seven crosses produced such plants: p35S:AtRALF1/*atsbt5.3* and p35S:AtRALF1/*atsbt6.1* (Fig. 2). Although the other crosses produced taller plants and longer primary roots than the p35S:AtRALF1 parental line, growth remained clearly compromised compared with p35S:AtRALF1/*atsbt5.3* and p35S:AtRALF1/*atsbt6.1*. The incomplete recovery of the p35S:AtRALF1 phenotype may reflect ectopic AtRALF1 expression driven by the 35S promoter. AtSBT6.1 has been implicated in processing AtRALF23 (Srivastava et al. 2009). Because AtSBT6.1 is expressed in nearly all Arabidopsis tissues, this protease may process not only AtRALF23 but also other isoforms (Abarca et al. 2021), including AtRALF1.

Genes driven by the 35S promoter are generally considered constitutive and expressed in most cell types (Benfey and Chua 1990). The strong and ectopic expression of AtRALF1 in p35S:AtRALF1-mCherry plants may explain why AtSBT6.1 and AtSBT5.3 function together in precursor processing under overexpression conditions. In our mutant analysis, *atsbt6.1* showed longer PRL and higher LRP density than wild-type plants, whereas *atsbt5.3* showed PRL and LRP density values like those of wild-type plants (Fig. 3A, B). The only subtilase mutant with responses consistent with impaired endogenous AtRALF1 processing was *atsbt6.1*, suggesting that AtSBT6.1 plays a predominant role in AtRALF1 precursor processing when AtRALF1 is expressed from its native promoter. However, loss of *AtSBT5.3* caused complete reversal of the LRP phenotype and partial reversal of the PRL phenotype in p35S:AtRALF1-mCherry/*atsbt5.3* descendants. We therefore conclude that AtSBT5.3 can contribute to preproAtRALF1 processing in tissues such as the epidermis, where AtRALF1 is ectopically expressed from the 35S promoter. Sénéchal et al. (2014) showed that PME17 processing involves specifically co-expressed AtSBTs depending on tissue context. AtSBT5.3 may also participate in processing other AtRALF precursors, such as preproAtRALF34, an important regulator of lateral root initiation expressed in the epidermis (Murphy et al. 2016; Gonneau et al. 2018).

Although preproAtRALF1 appears to be processed primarily by AtSBT6.1 in vivo, some peptide precursors are processed by two or more subtilases, such as IDA precursor which is processed by three different AtSBTs, and they may act redundantly in IDA maturation (Schardon et al. 2016). In the same way, the GOLVEN1 precursor is processed by AtSBT6.1 and its close homolog AtSBT6.2 (Ghorbani et al. 2016) and CLE40 and SCOOP20 precursors are processed in the same cleavage site by multiple subtilases (Stührwohldt et al. 2020b; Yang et al. 2023). Subtilases may exhibit flexible substrate specificity, recognizing extended sequence motifs around the cleavage site rather than a single amino acid residue, and the protease–substrate relationships in peptide signaling are not always one-to-one; because processing specificity may depend on co-expression, tissue context, subcellular localization and substrate abundance (Matsui and Hirakawa 2026). Such functional redundancy may help explain why AtSBT6.1 appears to be sufficient for AtRALF1 processing under endogenous expression, whereas AtSBT5.3 can participate when *AtRALF1* is overexpressed. The absence of an obvious phenotype, as observed for *atsbt5.3*, is frequent in single-gene loss-of-function subtilase mutants, likely because of functional redundancy among subtilases (Rautengarten et al. 2005). While these results support the involvement of AtSBT6.1 and AtSBT5.3 in AtRALF1 precursor processing, they do not establish that either subtilase directly cleaves the AtRALF1 precursor. Therefore, an indirect mechanism involving regulation of, or interaction with, other protease(s) responsible for AtRALF1 maturation cannot be excluded.

Microsomal fractions contain membranes derived from several components of the endomembrane system, including the plasma membrane, endoplasmic reticulum, Golgi apparatus, vacuolar membranes and endosomal compartments (Abas and Luschnig 2010). The ER and Golgi apparatus are particularly relevant for post-translational processing and glycosylation. AtSBT6.1, but not AtSBT5.3, has previously been localized to the Golgi apparatus (Liu et al. 2007), and its proteolytic activity has been associated with microsomal fractions that include Golgi vesicles (Wolf et al. 2009). For example, CLEL6 precursor involves sequential processing, where the SBT6.1 catalyzes the initial cleavage event in the early Golgi compartment, followed by N-terminal cleavage and final activation by SBT3.8 within the acidic post-Golgi secretory compartments, including the apoplast space (Stührwohldt et al. 2020a, b). Our results demonstrate that, after entering the ER, AtRALF1 preproprotein is processed by subtilases in the Golgi complex, considered not only a trafficking station but also a functional compartment where the inactive AtRALF1 precursor is converted into a bioactive ligand. Based on the subcellular localization of AtSBT6.1 and AtSBT5.3 (Supplementary Fig. S6A–F), we propose that both proteins may contribute to AtRALF1 preprotein processing under conditions in which substrate availability and tissue expression overlap.

Recent studies have shown that RALF peptide signaling integrates extracellular pH regulation, cell wall sensing and immune signaling through dynamic interactions among FERONIA, LRX proteins and other receptor-like kinases (Cheung and Wu 2024). In this context, precursor processing may represent an additional regulatory layer controlling ligand availability, receptor specificity and downstream signaling intensity. Consequently, tissue-specific expression patterns of subtilases such as AtSBT6.1 and AtSBT5.3 may contribute to the functional diversification of RALF signaling pathways in different biological scenarios.

Emerging evidence indicates that RALF-mediated signaling also plays important roles in plant-pathogen interactions. Several pathogens exploit RALF-like peptides or manipulate FERONIA signaling to promote infection and suppress immune responses (Zhang et al. 2020; Chen et al. 2025). Therefore, understanding how endogenous RALF peptides are proteolytically activated may contribute not only to developmental biology but also to future strategies for improving plant resistance and stress tolerance. Taken together, our results support a model in which AtSBT6.1 plays the predominant role in physiological AtRALF1 precursor processing, whereas AtSBT5.3 contributes under ectopic overexpression conditions. This functional specialization likely reflects differences in tissue-specific expression, substrate accessibility and secretory-pathway dynamics.

## Supplementary Information

Below is the link to the electronic supplementary material.

Supplementary file1 (PPTX 12946 KB)

## Data Availability

The datasets generated and analyzed during the current study are available from the corresponding author upon reasonable request. Raw data supporting the figures should be deposited or provided as supplementary material before submission.
